# Comparison of Three Evaluation Methods for the Lumbar Intervertebral Disc Vacuum Phenomenon in Predicting Postoperative Residual Lower Back Pain

**DOI:** 10.7759/cureus.93408

**Published:** 2025-09-28

**Authors:** Shuhei Ohyama, Yasuchika Aoki, Masahiro Inoue, Yusuke Sato, Masashi Sato, Hiromasa Wakita, Satoshi Yoh, Hiroshi Takahashi, Arata Nakajima, Toshiaki Kotani, Eguchi Yawara, Sumihisa Orita, Kazuhide Inage, Yasuhiro Shiga, Koichi Nakagawa, Seiji Ohtori

**Affiliations:** 1 Orthopedic Surgery, Eastern Chiba Medical Center, Togane, JPN; 2 Orthopedic Surgery, Graduate School of Medicine, Chiba University, Chiba, JPN; 3 General Medical Science, Graduate School of Medicine, Chiba University, Chiba, JPN; 4 Orthopedic Surgery, Institute of Medicine, University of Tsukuba, Tsukuba, JPN; 5 Orthopedic Surgery, Toho University Sakura Medical Center, Sakura, JPN; 6 Orthopedic Surgery, Seirei Sakura Citizen Hospital, Sakura, JPN

**Keywords:** low-back pain (lbp), predictor tools, residual pain, transforaminal lumbar interbody fusion (tlif), vacuum disc

## Abstract

Introduction: The lumbar intervertebral disc vacuum phenomenon (VP) is an important imaging finding commonly seen in patients with lumbar degenerative disease (LDD). It is closely associated with low back pain (LBP) and surgical outcomes after spine surgery. This study aims to compare different evaluation methods of VP to determine which method best predicts postoperative residual LBP after single-level transforaminal lumbar interbody fusion (TLIF) for LDD.

Methods: Overall, 66 patients (67.8 ± 10.2 years; 36 male/30 female) with LDD treated by single-level TLIF, who showed preoperative LBP, were enrolled in this study. The severity of VP (SVP) score was evaluated by three methods: SVP1 score, Willhuber’s classification; SVP2 score, a modified classification excluding subchondral sclerosis; and SVP3 score, presence/absence of VP. Clinical outcomes were assessed. Patients were divided into the residual LBP group (R group) and the control group (C group) by postoperative visual analogue scale (VAS) for LBP. Logistic regression analyses (both univariate and multivariable adjusted for age, sex, body mass index, lumbar lordosis, and pelvic incidence minus lumbar lordosis) were performed with the presence of residual LBP as the dependent variable and each SVP score as an independent variable. Receiver operating characteristic (ROC) curves and the area under the curve (AUC) were used to evaluate predictive ability, and DeLong’s test was used to compare AUCs.

Results: Each SVP score was significantly greater in the R group than in the C group. There were no significant differences in preoperative clinical outcomes. The AUCs of SVP1, SVP2, and SVP3 were 0.789, 0.802, and 0.788, respectively. Although SVP2 had the largest AUC, DeLong’s test showed no statistically significant differences among the three methods. In multivariable analyses, all SVP scores remained significant predictors of residual LBP.

Conclusion: Evaluation of VP severity based on VP size (modified Willhuber’s classification, SVP2) demonstrated the highest numerical predictive accuracy for postoperative residual LBP, but differences compared with other methods were not statistically significant. SVP scoring may serve as a useful preoperative tool.

## Introduction

Lumbar degenerative disease (LDD) is characterized by lower back pain (LBP) and lower extremity symptoms, including intermittent claudication (IC) [1,2]. Good results have been reported for transforaminal lumbar interbody fusion (TLIF) in patients with LDD [3]. In contrast, residual symptoms, especially residual LBP, may occur in patients with LDD after TLIF, which is an important issue related to the quality of life in patients with LDD [4]. Various factors, such as age and spinopelvic parameters, contribute to the postoperative residual LBP [5,6]. Postoperative residual LBP is a distinct clinical concern that differs mechanistically from other complications such as infection, hardware failure, or adjacent segment disease. It often results from pain originating in unfused but degenerated discs or spinal imbalance, which are not fully addressed by single-level fusion. Unlike radicular symptoms, which tend to improve after nerve decompression, residual LBP reflects persistent axial pain that is less responsive to standard surgical interventions. Preoperative identification of patients prone to residual LBP is valuable for improving patient counseling and managing expectations.

The lumbar intervertebral disc vacuum phenomenon (VP) is an important imaging finding that suggests severe disc degeneration [7]. VP is a gas formed within the intervertebral disc [7,8]. It is closely associated with LBP and surgical outcomes in patients with LDD after spine surgery [9]. Willhuber et al. developed a classification system to evaluate the severity of VP (SVP) based on the size of the VP and the subchondral sclerosis of the endplate [10]. This classification is based on a study to determine the indications of discoplasty for degenerative disc disease; however, the SVP based on Willhuber’s classification is reportedly associated with surgical outcomes after single-level TLIF [11,12]. However, no classification of the SVP has focused on the postoperative residual LBP, and the appropriate method for the evaluation of VP to predict postoperative residual LBP is not well known. Some components of existing classifications, such as subchondral sclerosis, may have limited relevance to postoperative residual LBP. Subchondral sclerosis is thought to be a compensatory change rather than a direct source of pain, and its contribution to mechanical pain generation remains unclear. In addition, a cut-off value for the SVP to predict the postoperative residual LBP after single-level TLIF has not been established.

This study aims to explore the appropriate method for the evaluation of VP to predict the postoperative residual LBP and to determine the cut-off value for the SVP to predict the postoperative residual LBP after single-level TLIF for LDD.

## Materials and methods

Patients

This study examined the clinical records of 134 patients diagnosed with LDD, including those with lumbar degenerative spondylolisthesis, lumbar spinal stenosis, lateral lumbar disc herniation, lumbar foraminal stenosis, lumbar spondylolytic spondylolisthesis, and lumbar facet joint cysts, who were treated with single-level TLIF with or without additional decompression surgery at any other level of the lumbar spine at a single institution between May 2014 and October 2019. Spine surgeons with more than 10 years of clinical experience conducted all the procedures. All patients were followed for at least two years. Patients showing signs of other pathologies, such as infections, cancer, significant past trauma, and conditions impacting daily life (e.g., severe cardiopulmonary disease, dementia, or systemic inflammatory disorders), were excluded. Patients who underwent lumbar fusion surgery prior to the index procedure, as well as those who required reoperation within two years due to complications such as infection, implant failure, or adjacent segment disease, were also excluded. To enroll patients with preoperative low back pain (LBP), we included patients whose preoperative LBP visual analogue scale (VAS) score was ≥ 40 [13]. Patients were not excluded based on the presence or dominance of lower extremity symptoms (e.g., radiculopathy), as long as the LBP VAS threshold was met. Therefore, the study population may include patients with both axial and radicular pain symptoms. Of the 134 patients in this study, 28 met the exclusion criteria. Of the 106 patients, 40 had a preoperative VAS score for LBP of < 40.

Ethics statement

The study adhered to the guidelines of the Declaration of Helsinki, and the study protocol was approved by the Institutional Review Board of our hospital (Eastern Chiba Medical Center, Approval No. 68). Informed consent was obtained from all the patients.

Data collection

Patient demographic data, including age, sex, body mass index (BMI), and fusion level, were retrospectively analyzed. Spinopelvic parameters, including lumbar lordosis (LL; the angle between the superior endplates of L1 and S1) and pelvic incidence (PI; the angle between a line perpendicular to the sacral plate at its midpoint and the line from the center of the femoral head to the sacral endplate midpoint), were measured using preoperative lateral lumbar spine radiographs. Preoperative lumbopelvic imbalance was evaluated using the PI-LL value, and LL was measured two years after surgery. Preoperative lumbopelvic imbalance was evaluated using the PI-LL value, as it is a well-established parameter that influences postoperative outcomes in lumbar fusion surgery. Because our focus was on assessing the impact of preoperative sagittal alignment on residual LBP, PI-LL was measured only preoperatively.

The SVP score was calculated as the sum of the VP scores of the lumbar intervertebral discs from L1-L2 to L5-S1 (or L6-S1) other than the fused disc, using three evaluation methods based on CT reconstructed images. All VP evaluations were performed by two board-certified spine surgeons, each with over 10 years of clinical experience. Each VP score was assessed once by each rater. Intra-observer variability was not assessed, and the final VP type was determined through consensus if discrepancies arose. Evaluations were conducted independently using sagittal CT images, and discrepancies were resolved by consensus discussion. Although interobserver or intraobserver reliability was not formally assessed in this study, the scoring methods, particularly the Willhuber classification, have been used in prior studies and demonstrated acceptable reproducibility. 

VP scores were evaluated using three different classification methods. The SVP1 score was based on the original method described by Willhuber et al., which assigns scores from 0 to 5 based on the distribution of air in the intervertebral disc space and the presence or absence of subchondral sclerosis [10]. The scoring is defined as follows: Score 5: air in the disc space, in contact with both endplates, with subchondral sclerosis; Score 4: air in the disc space, in contact with both endplates, without subchondral sclerosis; Score 3: air in the disc space, in contact with one endplate, with subchondral sclerosis; Score 2: air in the disc space, in contact with one endplate, without subchondral sclerosis; Score 1: small amount of intervertebral air, without subchondral sclerosis; Score 0: no vacuum phenomenon.

SVP2 score, a novel modification developed for this study, which excludes the subchondral sclerosis component to focus solely on vacuum size (0 to 3 points) (Table 1). This modification was based on the hypothesis that subchondral sclerosis may represent a compensatory, non-pain-generating change, and therefore may not contribute meaningfully to predicting postoperative residual LBP. SVP3 score, a simplified method considering only the presence or absence of VP (0 to 1 point).

**Table 1:** Three methods of evaluation of the SVP score SVP score is the sum of the severity of the intervertebral vacuum phenomenon score of the five (or six) discs from L1-L2 to L5-S1 (or L6-S1) [10]. SVP: severity of vacuum phenomenon

Classification	Description	Score
SVP2	Air in the disc space, with air in contact with both endplates	3
	Air in the disc space, with air in contact with one endplate	2
	Small amount of intervertebral air, without subchondral sclerosis	1
	No vacuum phenomenon	0
SVP3	Air in the disc space	1
	No vacuum phenomenon	0

**Figure 1:**
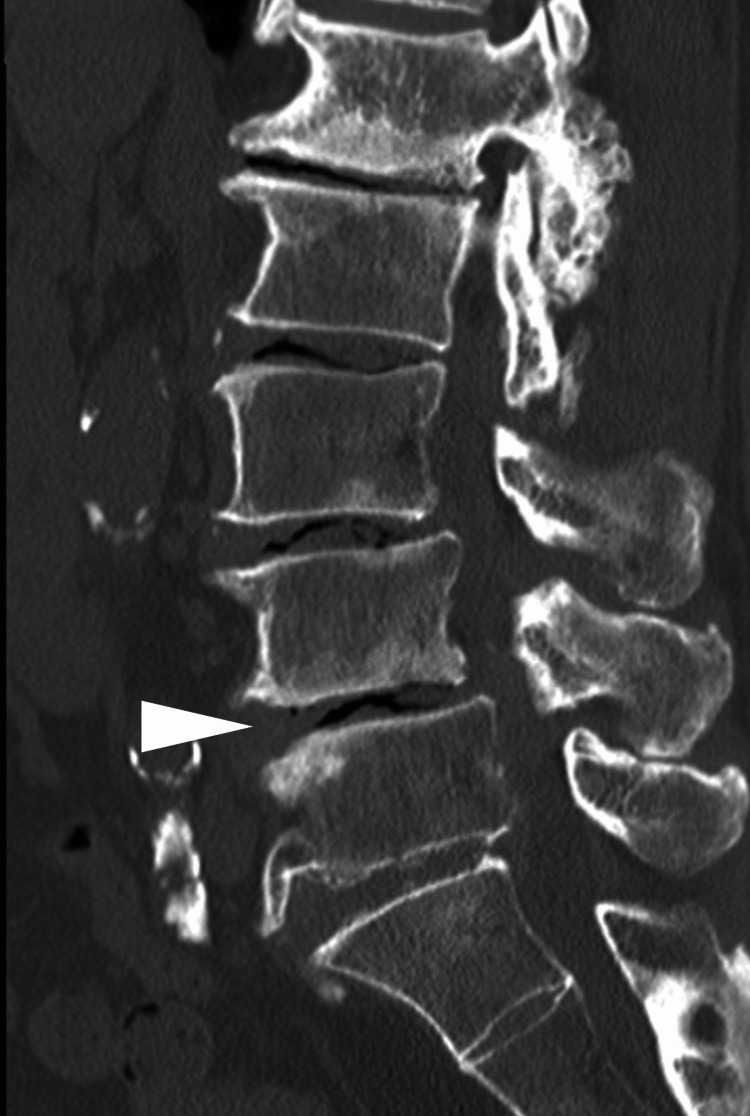
The preoperative midsagittal computed tomography of the lumbar spine The white arrowhead indicates the fusion level. In this image, SVP1 = 12, SVP2 = 8, and SVP3 = 3. SVP: severity of vacuum phenomenon

These three methods were selected to compare the predictive value of different evaluation approaches, from comprehensive (SVP1), to size-focused (SVP2), to binary presence (SVP3) (Table 1). VP scores were evaluated based on sagittal CT images (right parasagittal, middle sagittal, and left parasagittal). The vacuum type that prevailed in two of the three CT images determined the final vacuum type [10]. A representative example is shown in Figure 1.

Clinical outcomes were assessed using the VAS for LBP, lower extremity pain, and numbness; this scoring system was used for LBP in motion, as well as in the standing and sitting positions [14]; the Japanese Orthopaedic Association Back Pain Evaluation Questionnaire (JOABPEQ) [15-20]; the Japanese Orthopaedic Association Score for IC (JOA score for IC); and Nakai’s scoring system for the evaluation of surgical outcome, with scores ranging from 3 (excellent) to 0 (poor) [18]. Clinical data were evaluated preoperatively and two years after the surgery.

Comparison between patients with and without postoperative residual lower back pain

To compare patients with and without postoperative LBP, patients with an LBP-VAS score >25 at two years postoperatively were assigned to the R group (residual LBP group), and all other patients were assigned to the C group (control group), based on the previously reported patient acceptable symptom state threshold for postoperative LBP [13]. Of the 66 patients, 16 and 50 were assigned to the R and C groups. Patient data, spinopelvic parameters, and preoperative clinical outcomes were compared between the R and C groups.

Comparison of the three evaluation methods for the severity of the vacuum phenomenon score

Logistic regression analysis was performed with the presence of postoperative residual LBP (LBP-VAS scores > 25 at two years postoperatively) as a dependent variable and SVP1, SVP2, and SVP3 scores as independent variables. The receiver operating characteristic (ROC) curve for each variable was described, and the area under the curve (AUC) was calculated. The cut-off value for the SVP score predicting postoperative residual LBP was calculated using the Youden index based on the model with the best AUC of the three assessment methods.

Comparison of patient data based on the best evaluation methods for the severity of the vacuum phenomenon score

To confirm the accuracy of the best evaluation methods for SVP scores, patient data were compared by dividing the patients into two groups based on the cut-off value of the best evaluation methods for the SVP score.

Data analysis

Continuous variables (age, BMI, spinopelvic parameters, SVP scores, VAS, JOA score for IC, and Nakai’s score) were compared using an unpaired t-test, categorical variables (sex, diagnosis of surgery, and fusion level) were compared using a chi-square test, and non-normally distributed variables (JOABPEQ scores) were compared using a Mann-Whitney U test. Comparisons were performed between the R group and the C group. Standardized mean differences (SMDs; Cohen’s d) were calculated for continuous variables to provide effect size estimates and facilitate comparability between the two groups. Univariate logistic regression analysis was performed using each SVP score (SVP1, SVP2, and SVP3) as the independent variable and the presence of postoperative residual LBP as the dependent variable. Subsequently, multivariable logistic regression was performed for each SVP score, adjusting for age, sex, BMI, preoperative PI-LL, and preoperative LL as potential confounders. Results are presented as adjusted odds ratios (aOR) per 1-point increase with 95% confidence intervals (CIs). Discrimination was assessed with the AUC. To statistically compare the AUCs between the three scoring methods (SVP1, SVP2, and SVP3), DeLong’s test for two correlated ROC curves was used [21]. Statistical significance was set at p < 0.05. Values were expressed as the mean ± standard deviation (SD). The JOABPEQ values were expressed as median values (maximum-minimum).

## Results

Of the 134 patients in this study, 28 met the exclusion criteria. Of the 106 patients, 40 had a preoperative VAS score for LBP of < 40. Finally, 66 patients (67.8 ± 10.2 years; 36 male and 30 female; BMI: 25.2 ± 3.5 kg/m^2^) were enrolled in this study. The patient data are shown in Table 2.

**Table 2:** Patient data used in this study Data are presented as the mean ± standard deviation.

Parameters		Variables
Age (year)		67.8 ± 10.2
Sex (male/female)		36/30
Body mass index (kg/m^2^)		25.2 ± 3.5
Diagnosis for surgery	Degenerative spondylolisthesis	40
	Spinal stenosis	10
	Lateral disc herniation	4
	Foraminal stenosis	6
	Spondylolytic spondylolisthesis	5
	Facet joint cyst	1
Fusion segment	L3-4	5
	L4-5	46
	L5-6	1
	L5-S	14
Additional decompression surgery		22

Comparison between patients with and without postoperative residual low back pain

Of the 66 patients, 16 and 50 were assigned to the R and C groups, respectively. There were no significant differences in the patient demographic data and spinopelvic parameters (Table 3). As shown in Table 3, each SVP score was significantly higher in the R group than in the C group. There were no significant differences in the preoperative clinical outcomes (Table 3).

**Table 3:** Comparison between patients with and without postoperative residual low back pain Data are presented as the mean ± standard deviation. Asterisks (*) indicate statistically significant differences (p-value < 0.05). R group: residual low back pain group; C group: control group; SMD: standardized mean differences; LL: lumbar lordosis; PI: pelvic incidence; SVP: severity of vacuum phenomenon; SVP score is the sum of the severity of the intervertebral vacuum phenomenon score of the five (or six) discs from L1-L2 to L5-S1 (or L6-S1); VAS: visual analogue scale; JOABPEQ: The Japanese Orthopaedic Association Back Pain Evaluation Questionnaire; JOA score for IC: Japanese Orthopaedic Association score for intermittent claudication; 3 = normal to 0 = unable to walk > 100 m

Parameters		R group	C group	p-value	Test statistic	SMD
Number of patients		16	50	-	-	-
Age (years)		72.8 ± 8.0	66.2 ± 10.4	0.014*	t = 2.59	0.66
Sex (male/female)		7/9	29/21	0.32	ꭓ^2 ^= 1.00	-
Body mass index (kg/m^2^)		25.5 ± 2.4	25.1 ± 3.8	0.22	t = -1.24	0.11
Diagnosis for surgery	Degenerative spondylolisthesis	8	32	0.79	ꭓ^2 ^= 2.38	-
	Spinal stenosis	4	6			
	Lateral disc herniation	1	3			
	Foraminal stenosis	2	4			
	Spondylolytic spondylolisthesis	1	4			
	Facet joint cysts	0	1			
Fusion segment	L3-4	1	4	0.88	ꭓ^2 ^= 0.66	-
	L4-5	11	35			
	L5-6	0	1			
	L5-S	4	9			
Additional decompression surgery		8	14	0.10	ꭓ^2 ^= 2.65	-
Preoperative LL (°)		36.0 ± 15.2	37.7 ± 12.8	0.70	t = -0.39	-0.12
Preoperative PI (°)		50.9 ± 6.6	51.4 ± 9.8	0.83	t = 0.27	-0.05
Preoperative PI-LL (°)		9.9 ± 15.3	13.8 ± 13.7	0.79	t = -0.22	0.08
Postoperative LL (°)		34.5 ± 19.2	39.1 ± 11.4	0.39	t = -0.89	-0.33
LL restoration (°)		-1.5 ± 6.6	1.5 ± 10.1	0.20	t = -1.32	-0.31
Preoperative SVP1 score		11.9 ± 6.5	4.4 ± 5.5	0.001*	t = 3.69	1.17
Preoperative SVP2 score		7.2 ± 3.9	2.7 ± 3.4	<0.001*	t = 4.03	1.27
Preoperative SVP3 score		2.7 ± 1.4	1.1 ± 1.2	<0.001*	t = 3.97	1.23
Preoperative VAS	Lower back pain (LBP)	74.5 ± 12.0	73.9 ± 16.3	0.87	t = 0.16	0.04
	Lower extremity pain	77.3 ± 13.7	74.2 ± 22.2	0.53	t = 0.64	0.15
	Lower extremity numbness	76.7 ± 21.8	73.7 ± 24.6	0.68	t = 0.42	0.12
Preoperative detailed VAS	LBP in motion	53.8 ± 26.5	58.9 ± 30.4	0.54	t = -0.62	-0.17
	LBP in standing	71.6 ± 17.6	73.5 ± 24.5	0.73	t = -0.34	-0.08
	LBP in sitting	61.1 ± 18.1	52.8 ± 25.3	0.17	t = 1.69	0.35
Preoperative JOABPEQ	Pain-related disorders	21.5 (100-0)	29 (100-0)	0.51	U = 348.5	-
	Lumbar spine dysfunction	54 (100-17)	42 (100-0)	0.097	U = 491.0	-
	Gait disturbance	29 (86-0)	21 (93-0)	0.79	U = 368.5	-
	Social life disturbance	46 (57-3)	32 (73-0)	0.29	U = 442.5	-
	Psychological disorders	40 (78-9)	45 (76-3)	0.58	U = 339.0	-
Preoperative JOA score for IC		0.7 ± 0.6	0.8 ± 0.9	0.59	t = 0.55	0.15

Multivariable analysis

After adjustment for age, sex, BMI, preoperative PI-LL, and preoperative LL, all three SVP scores remained significantly associated with postoperative residual LBP (Table 4).

**Table 4:** Multivariable logistic regression analysis for postoperative residual LBP (adjusted for age, sex, BMI, preoperative PI–LL, and preoperative LL) Asterisks (*) indicate statistically significant differences (p-value < 0.05). LBP: low back pain; BMI: body mass index; PI: pelvic incidence; LL: lumbar lordosis; OR: odds ratio; CI: confidence interval; SVP: severity of vacuum phenomenon; SVP score is the sum of the severity of the intervertebral vacuum phenomenon score of the five (or six) discs from L1-L2 to L5-S1 (or L6-S1); Preop: preoperative

Predictor	Adjusted OR	95% CI	p-value
SVP1	1.22	1.03 – 1.42	0.001*
Age (years)	1.05	0.96 – 1.16	0.27
Sex (male vs female)	0.39	0.07 – 1.78	0.23
BMI (kg/m²)	1.10	0.89 – 1.37	0.36
Preop PI–LL (°)	0.94	0.85 – 1.03	0.20
Preop LL (°)	0.99	0.89 – 1.09	0.82
SVP2	1.43	1.17 – 1.85	<0.001*
Age (years)	1.05	0.96 – 1.16	0.28
Sex (male vs female)	0.36	0.06 – 1.70	0.20
BMI (kg/m²)	1.10	0.88 – 1.38	0.38
Preop PI–LL (°)	0.95	0.84 – 1.03	0.20
Preop LL (°)	0.99	0.89 – 1.10	0.86
SVP3	2.98	1.61 – 6.51	<0.001*
Age (years)	1.05	0.96 – 1.16	0.30
Sex (male vs female)	0.29	0.05 – 1.43	0.13
BMI (kg/m²)	1.11	0.89 – 1.39	0.34
Preop PI–LL (°)	0.93	0.84 – 1.02	0.11
Preop LL (°)	0.98	0.88 – 1.08	0.69

Comparison of three evaluation methods for the severity of the vacuum phenomenon score

**Figure 2:**
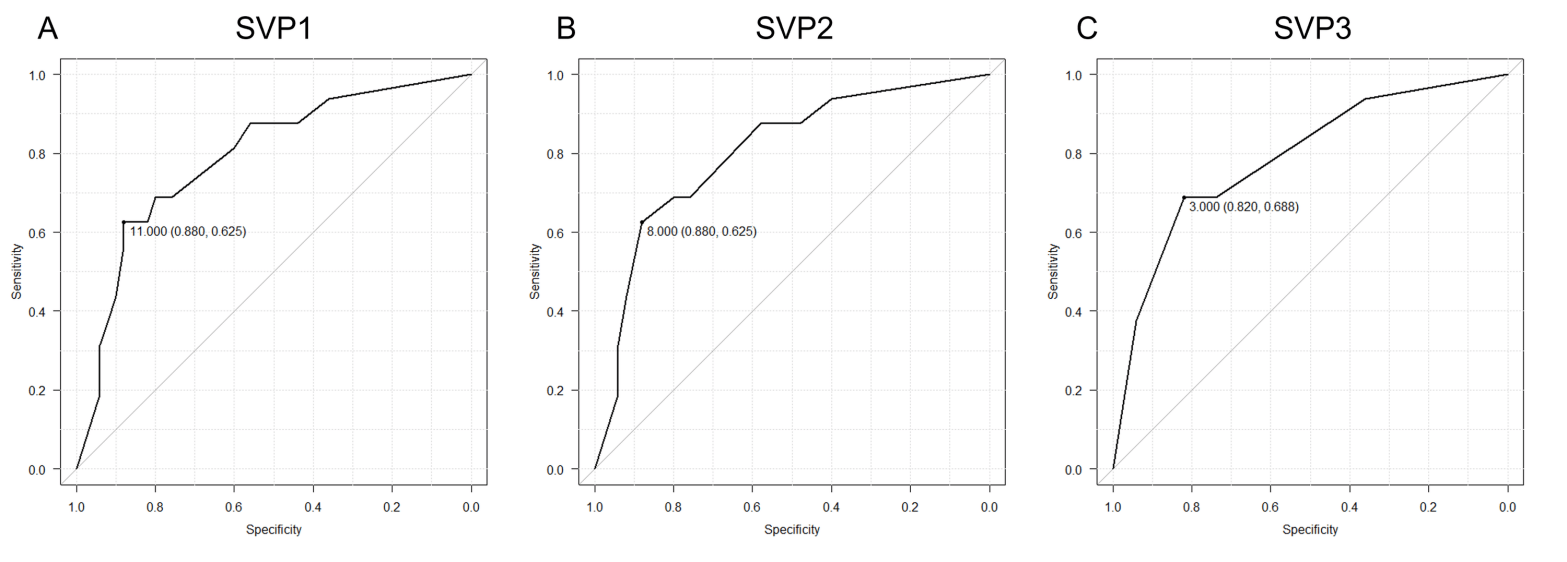
Receiver operating characteristic curves for the three evaluation methods for the SVP score A: SVP1; B: SVP2; C: SVP3 SVP: severity of vacuum phenomenon

The ROC curves for the three evaluation methods for the SVP scores are shown in Figure 2. The AUCs for SVP1, SVP2, and SVP3 were 0.789, 0.802, and 0.788, respectively, with the SVP2 score having the largest AUC. The cut-off value for residual LBP in the SVP2 score using the Youden Index was 8.0, with a sensitivity of 0.63 and specificity of 0.88 (Figure 2). DeLong’s test revealed no statistically significant differences between the AUCs of the three scoring methods. The AUC difference between SVP1 and SVP2 was −0.01 (Z = −0.25, p = 0.80), between SVP1 and SVP3 was 0.00 (Z = 0.02, p = 0.98), and between SVP2 and SVP3 was 0.02 (Z = 0.27, p = 0.79).

Comparison of outcomes when patients were divided into two groups at the cut-off value (SVP2 = 8)

Patients were divided into two groups based on SVP2 (= 8); the severe (SVP2 ≥ 8) (n = 16) and mild VP group (SVP2 < 8) (n = 50). As shown in Table 5, patients in the severe VP group were significantly older, but the preoperative clinical outcomes were similar, except for the VAS score for lower extremity numbness. However, the patients in the severe VP group had significantly worse VAS, JOABPEQ, and Nakai’s scores, except for the VAS score for LBP in the sitting position.

**Table 5:** Comparison of outcomes when patients were divided into two groups at the cutoff value (SVP2 = 8) Data are presented as the mean ± standard deviation. Asterisks (*) indicate statistically significant differences (p-value < 0.05). SVP: severity of vacuum phenomenon; SVP score is the sum of the severity of the intervertebral vacuum phenomenon score of the five (or six) discs from L1-L2 to L5-S1 (or L6-S1); SMD: standardized mean differences; LL: lumbar lordosis; PI: pelvic incidence; Preop: preoperative; Postop: postoperative; VAS: visual analogue scale; JOABPEQ: The Japanese Orthopaedic Association Back Pain Evaluation Questionnaire; JOA score for IC: Japanese Orthopaedic Association score for intermittent claudication; 3 = normal to 0 = unable to walk >100 m; Nakai’s score: 3 = excellent to 0 = poor

Parameters			Severe VP group (SVP2 score≧8)	Mild VP group (SVP2 score<8)	p-value	Test statistic	SMD
Number of patients			16	50	-	-	-
Age (years)			72.2 ± 9.1	66.4 ± 10.2	0.044*	t = 2.11	0.58
Sex (male/female)			10/6	26/24	0.17	ꭓ^2 ^= 0.20	-
Body mass index (kg/m^2^)			25.4 ± 2.7	25.1 ± 3.7	0.80	t = 0.26	0.06
Preoperative LL (°)			30.6 ± 13.7	39.4 ± 12.6	0.038*	t = -2.20	-0.67
Preoperative PI (°)			50.8 ± 6.4	51.5 ± 9.8	0.74	t = -0.34	-0.08
Preoperative PI-LL (°)			20.1 ± 11.9	12.1 ± 13.8	0.036*	t = 2.20	0.59
Postoperative LL (°)			28.8 ± 17.1	41.0 ± 11.6	0.017*	t = -2.60	-0.94
LL restoration (°)			-1.9 ± 6.1	1.6 ±10.2	0.12	t = -1.61	-0.36
VAS	Lower back pain (LBP)	Preop	74.3 ± 14.8	73.9 ± 15.6	0.93	t = 0.09	0.03
		Postop	42.3 ± 30.8	14.4 ± 19.3	0.004*	t = 3.32	1.21
	Lower extremity pain	Preop	77.3 ± 15.5	74.1 ± 21.9	0.54	t = 0.62	0.15
		Postop	39.6± 29.0	13.6 ± 20.0	0.004*	t = 3.25	1.14
	Lower extremity numbness	Preop	84.4 ± 12.7	71.1 ± 25.8	0.010*	t = 2.69	0.56
		Postop	47.8 ± 27.3	14.3 ± 21.3	<0.001*	t = 4.36	1.44
Detailed VAS	LBP in motion	Preop	61.0 ± 26.3	56.5 ± 30.5	0.58	t = 0.55	0.15
		Postop	33.3 ± 25.6	15.0 ± 22.0	0.021*	t = 2.49	0.78
	LBP in standing	Preop	77.4 ± 18.8	71.6 ± 24.0	0.34	t = 0.96	0.25
		Postop	39.4 ± 24.7	14.1 ± 20.0	0.002*	t = 3.63	1.18
	LBP in sitting	Preop	57.9 ± 18.9	53.8 ± 25.4	0.50	t = 0.67	0.17
		Postop	25.4 ± 23.5	11.9 ± 18.5	0.055	t = 2.03	0.67
JOABPEQ	Pain-related disorders	Preop	29 (71-0)	29 (100-0)	0.63	U = 360.0	-
		Postop	71 (100-0)	100 (100-43)	<0.001*	U = 213.5	-
	Lumbar spine dysfunction	Preop	42 (100-0)	50 (100-0)	0.64	U = 353.5	-
		Postop	75 (100-17)	83 (100-42)	0.020*	U = 250.0	-
	Gait disturbance	Preop	17.5 (86-0)	21 (93-0)	0.34	U = 309.0	-
		Postop	50 (100-0)	93 (100-29)	0.002*	U = 182.0	-
	Social life disturbance	Preop	42 (57-0)	32 (70-0)	0.11	U = 399.0	-
		Postop	51 (100-30)	78 (100-51)	0.007*	U = 203.0	-
	Psychological disorders	Preop	40 (60-3)	46 (78-6)	0.16	U = 299.0	-
		Postop	48 (76-28)	63 (100-51)	0.004*	U = 189.5	-
JOA score for IC		Preop	0.7 ± 0.7	0.9 ± 0.9	0.36	t = -0.94	-0.24
		Postop	2.8 ± 0.4	2.7 ± 0.8	0.75	t = 0.32	0.07
Nakai’s score			2.1 ± 0.9	2.7 ± 0.6	0.023*	t = -2.47	-0.85

## Discussion

This study showed that the modified Willhuber’s classification (SVP2 score) is the most accurate predictor of VP and the degree of postoperative residual LBP. In addition, we show that the appropriate cut-off for the same was eight when measured using the SVP2 score.

In this study, the SVP2 score, which was evaluated by excluding the subchondral sclerosis of the endplate subtype from Willhuber’s classification, was the most useful predictor of postoperative residual LBP. In addition, multivariable logistic regression analyses adjusting for age, sex, BMI, preoperative PI-LL, and preoperative LL demonstrated that all three SVP scoring methods (SVP1, SVP2, SVP3) were independently associated with postoperative residual LBP (Table 4). Among these, the SVP2 model showed the highest discriminative ability, supporting the clinical relevance of VP size as a predictor. Although SVP2 had the highest numerical AUC, DeLong’s test revealed no statistically significant differences in AUCs among the three scoring methods (SVP1 vs. SVP2, SVP1 vs. SVP3, SVP2 vs. SVP3). This indicates that the improved discriminative ability of SVP2 over the others may not be statistically meaningful and should be interpreted with caution. Further validation in larger studies is warranted to confirm its utility. Several evaluations of the SVP have been reported; however, these classifications were made for different purposes, such as to determine the indications of discoplasty [10]. No VP classification has been developed to evaluate postoperative residual LBP. We proposed and compared three methods of evaluation for the prediction of postoperative residual LBP. The improved accuracy of the SVP2 score suggests that the presence or absence of the subchondral sclerosis of the endplate is not a useful predictor of LBP. The subchondral sclerosis of the endplate is generally considered a compensatory mechanism for the loss of the shock absorber function of the intervertebral disc and the increased burden on the vertebral body [19,20]. Therefore, it may not be directly associated with LBP. However, it has also been suggested that sclerosis may reflect the chronicity of degenerative changes, which could influence LBP differently in some patients. This possibility should be addressed in future investigations. In contrast, the improved accuracy of the SVP2 score as compared to the SVP3 score suggests that the size of the VP is an important predictor of its severity. A larger VP suggests a reduced shock absorber function of the intervertebral disc [19]. Therefore, the SVP2 score was more useful than the SVP3 score because it assessed VP size in more detail. From a biomechanical perspective, VP size is directly related to the loss of intervertebral disc integrity. A larger VP reflects greater gas accumulation within the disc space, which indicates reduced hydrostatic pressure and diminished shock absorption capacity of the disc. This loss of cushioning leads to increased load transfer to the vertebral endplates and facet joints, thereby accelerating degenerative changes and promoting segmental instability [19]. Segmental instability, in turn, has been strongly associated with persistent LBP after fusion procedures. Therefore, the superior predictive value of SVP2 in our study may be explained by its ability to capture VP size, which is a biomechanical surrogate for impaired shock absorption and increased spinal instability. The SVP is associated with factors related to postoperative residual symptoms, such as the degree of fatty infiltration of paraspinal muscles, the small LL, and the degree of disc degeneration [22-24]. In our study, the severe VP group exhibited a greater PI-LL mismatch compared with the mild VP group. This finding aligns with previous reports that multiple VPs in the lumbar discs are associated with reduced lumbar lordosis and sagittal imbalance. Such malalignment may exacerbate mechanical stress on adjacent segments and contribute to persistent LBP. These results suggest that VP severity not only reflects disc degeneration but may also be linked with global sagittal alignment, providing a potential mechanistic explanation for the association between SVP and poorer postoperative outcomes. Therefore, the SVP2 score may indirectly assess these related factors through the SVP. This is the first study to compare the methods of evaluation of VP and, thereby, to predict postoperative residual LBP and to show a cut-off value for the SVP score predicting postoperative residual LBP. A case with SVP2 ≧ 8 corresponds to a patient with a moderate VP in all lumbar intervertebral discs without a fused disc (SVP2 = 8) or those with severe VP in three intervertebral discs (SVP2 = 9). Spine surgeons can use this method of evaluation to help explain the procedure to patients before surgery.

Postoperative residual LBP in patients with LDD is critical for their quality of life [25]. In single-level TLIF, LBP can be improved by improving symptoms derived from a fused disc and poor lumbar flexion posture, with improvement in lower extremity symptoms [26,27]. On the other hand, it has been reported that the SVP other than in a fused disc affects surgical outcomes because symptoms derived from non-fused discs are difficult to improve [11,12]. In other words, the SVP in the lumbar spine as a whole, and not only locally, is important for postoperative residual LBP. In this study, lower extremity symptoms were worse in the severe VP group. Previous studies have reported that age, severity of symptoms, and duration of symptoms are important factors in postoperative residual lower-extremity symptoms [5,6]. The fact that patients in the severe VP group were older than those in the mild VP group supports these findings. The severe VP group may have had more severe lower-extremity symptoms; however, the lack of significant differences in the preoperative clinical assessment may have been due to the ceiling effect of the patient-oriented questionnaire [28]. Postoperative residual symptoms are known to persist in some patients; however, patients themselves hope that lumbar spine surgery will improve their LBP and lower extremity symptoms [29]. The degree of improvement of symptoms is an important factor in patient satisfaction after spine surgery [30]. The severe VP group showed worse surgical outcomes, including JOABPEQ and Nakai’s scores. In this study, the presence of severe VP affected not only postoperative LBP but also surgical satisfaction after single-level TLIF. The results of this study predicted the degree of postoperative residual LBP with high accuracy and contributed significantly to the preoperative explanations for patients. These findings may influence the choice of surgical strategy for patients predicted to have severe postoperative residual LBP. Patients with severe disc degeneration of the entire lumbar spine reportedly show smaller LL and may present with whole-spine malalignment, which is equivalent to an adult spinal deformity. Long spinal fusion can improve both the instability of intervertebral discs with VP and spinal malalignment and has been reported to be useful in the treatment of adult spinal deformities [31]. Therefore, long spinal fusion could be considered as a potential treatment option for patients with severe VP. However, this concept remains speculative. Long spinal fusion is highly invasive, has a high complication rate, and is not always indicated for patients with severe VP [32]. The threshold for selecting patients who might benefit from such an approach remains unknown, and prospective studies are required to validate this hypothesis.

Limitations

First, this method for evaluation of the VP is not the best method for predicting LBP; it is only the best among the three methods presented in this study. Second, this study did not identify any cause of postoperative residual LBP. It is well known that muscle mass, lumbar disc degeneration other than VP, and facet joint degeneration are related to LBP. These parameters are best evaluated with MRI, whereas our study relied on CT scans to assess VP only. Therefore, these items were not included in our analysis, and residual confounding cannot be excluded. Importantly, such degenerative markers may also influence surgical outcomes, meaning that their exclusion could have affected our findings. Nevertheless, this study suggests that VP scoring may serve as a useful preoperative tool despite these limitations. Third, the study population was heterogeneous. Patient pathophysiology and background data were heterogeneous in terms of the surgical diagnosis (e.g., lumbar spinal stenosis, degenerative spondylolisthesis, lateral lumbar disc herniation, foraminal stenosis, spondylolytic spondylolisthesis, and facet joint cyst), the level of fusion, and the presence or absence of additional decompression surgery. These factors may have influenced the results. Moreover, the severe VP group defined by SVP2 ≥ 8 included only 16 patients. This small subgroup size may limit the generalizability of the cut-off value, and larger studies are needed to validate its clinical applicability. Although there was no obvious heterogeneity in the distribution of diagnosis or fusion levels between the R and C groups, residual confounding cannot be excluded. Stratification or subgroup analyses by surgical diagnosis or fusion level could strengthen interpretability, but the sample size of this study was too limited to allow such analyses. Therefore, further studies with larger and more homogeneous cohorts are required to confirm these findings. Fourth, the interobserver and intraobserver reliability of VP scoring was not evaluated in this study. While the classification systems used have been previously validated, further assessment of scoring consistency would strengthen the methodology. Additionally, SVP scoring was performed once per evaluator, and intra-observer reliability was not evaluated. This may limit the reproducibility of our findings. Fifth, although age was adjusted for in the multivariable models, the residual imbalance in age between groups cannot be fully excluded as a source of confounding. More robust methods, such as propensity score matching, could address this issue, but were not feasible in our study due to the limited sample size. Finally, we did not perform a formal power analysis. Given the relatively small sample size and the limited number of patients in some subgroups, the possibility of type II error cannot be excluded. In particular, the number of patients with residual LBP was small (n = 16), which may affect the stability of the ROC curve analysis and limit the generalizability of the differences in AUC among the three scoring methods. Therefore, our findings should be interpreted with caution, and larger prospective studies with sufficient event numbers, power calculations, and more robust validation techniques are needed to confirm these results.

## Conclusions

In single-level TLIF for patients with LDD who have preoperative LBP, our results suggest that evaluating VP severity based on the size of VP (SVP2) may be a useful method for predicting postoperative residual LBP. However, this conclusion should be interpreted with caution, as it is derived from a single-center retrospective cohort with a limited sample size. External validation in larger, prospective, and multi-center studies is essential before the SVP2 method can be considered generalizable to broader clinical practice.
